# Assessment of the GNRB Arthrometer: High Inter‐Rater Reliability Alone Does Not Support Replacement of the KT1000 in Anterior Cruciate Ligament Reconstruction Patients

**DOI:** 10.1002/ars2.70056

**Published:** 2026-09-07

**Authors:** Sofia Lindqvist Bueneman, Jüri‐Toomas Kartus, Ninni Sernert

**Affiliations:** ^1^ Department of Orthopaedics Institute of Clinical Science Sahlgrenska Academy Gothenburg University Gothenburg Sweden; ^2^ Department of Orthopaedics NU Hospital Group Uddevalla Sweden; ^3^ Department of Research and Development NU Hospital Group Trollhättan Sweden

## Abstract

**Purpose:**

To evaluate the inter‐rater reliability and sensitivity of the GNRB arthrometer compared with the KT1000 and clinical examination with the Lachman test for patients with an anterior cruciate ligament (ACL) injury.

**Methods:**

Patients with ACL injury were examined preoperatively and postoperatively by 2 examiners. One examiner also performed a clinical examination and an examination with the KT1000. Side‐to‐side difference of ≥3 mm was classified as an ACL injury for both the GNRB and the KT1000. The interclass correlation was used to evaluate inter‐reliability, and a paired t‐test was used to identify differences between examiners. The sensitivity was calculated preoperatively.

**Results:**

A total of 20 patients underwent preoperative assessment with the GNRB, whereas 17 were available postoperatively. The GNRB diagnosed 12/20 and 13/20 for the 2 examiners, respectively, as having ACL injuries preoperatively, whereas the KT1000 diagnosed 19/20. Postoperatively, 5/17 and 4/17, respectively, had a side‐to‐side difference of ≥3 mm with the GNRB versus 7/17 for the KT1000, hence indicating an ACL injury or insufficient reconstruction. Clinical examination concluded that 15/17 had a manual Lachman of ≤+1. The interclass correlations between examiners were 0.80 for the involved side preoperatively and 0.84 postoperatively, considered moderate to good. Sensitivity for the GNRB was 60% and 65% for examiners 1 and 2, respectively. For the KT1000, it was 95%.

**Conclusions:**

In this study, the GNRB had a high inter‐rater reliability, even with limited experience with the device. However, it was inferior to the KT1000 in correctly identifying ACL deficiency preoperatively in patients with ACL injury.

**Level of Evidence:**

Level III, prospective comparative study.

## Graphical abstract

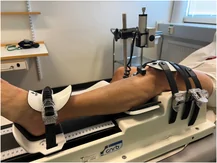

Rupture of the anterior cruciate ligament (ACL) is a traumatic injury to the knee that increases the laxity and increases the risk of osteoarthritis.^1^ Although a clinical examination offers reliability, especially on the injured side,^2^ it is also well known that the ability to set a correct diagnosis differs greatly among clinicians.^3^ To objectify these methods, several devices have been developed to mimic the manual Lachman test and give the anterior tibial translation a numerical value. The most common arthrometers on the market are the KT1000 (MEDmetric, San Diego, CA), Telos (Gmbh, Hungen/Obbornhafen, Germany), Rolimeter (Aircast, Summit, NJ), and the GNRB (Genourob, Laval, France). The latter has been available since 2005, marketed as the only device to offer reliable results in both full and partial tears of the ACL.^4^ Automatizing several steps, the GNRB differs from the earlier arthrometers used and has minimized the impact of the examiner.^4^ Several studies have found the GNRB more reliable and reproducible compared with the Telos and KT1000,^5^, ^6^, ^7^, ^8^ whereas others have questioned certain factors, such as patellar pressure load^9^, ^10^ and placement of the patellar pad.^11^ To obtain good reproducibility, the developers have suggested an anterior pressure load of 200 N to diagnose an ACL injury,^4^, ^12^ which is higher than the 134 N typically used with the KT1000.

Of the devices, the KT1000 is the most frequently used because of its simplicity and has been considered the gold standard together with the clinical examination. As the KT1000 has been discontinued, a good replacement device should serve as a helpful diagnostic and quantifying tool. Some studies have found that the KT1000 and GNRB are the most comparable,^7^, ^11^, ^13^ but the GNRB has higher reproducibility. Further, it offers both side‐to‐side difference (SSD) and slopes of laxity curves to evaluate the elasticity of the ACL. A threshold of 3 mm SSD for complete tears has been suggested in previous studies.^4^, ^14^

The purpose of the study was to evaluate the inter‐rater reliability and sensitivity of the GNRB arthrometer compared with the KT1000 and clinical examination with the Lachman test for patients with an ACL injury. We hypothesized that the GNRB offers high inter‐reliability and reproducibility before and after surgery and can replace the KT1000 in a clinical setting.

## METHODS

### Patients

Eligible patients were screened between August 2020 and August 2021 at the Orthopaedic Department of NU Hospital Group in Sweden. Inclusion criteria were 18 to 65 years of age and a magnetic resonance imaging or clinically verified first‐time ACL injury with a healthy contralateral knee. Patients with concomitant meniscal and/or cartilage injuries or grade 1 collateral ligament injuries were included. Exclusion criteria were <18 or >65 years of age, previous surgeries to any knee, posterior cruciate ligament injuries, or >grade 1 collateral ligament injuries.

Indications for surgery were determined based on joint instability, giveaway phenomena, athletic status of the patient, and perceived instability despite adequate rehabilitation. This was determined by the operating surgeon in accordance with the clinic's standard protocol.

During surgery, a diagnostic arthroscopy was performed to identify all injuries. Concomitant meniscal and/or cartilage injuries were treated at the surgeon's preference. All patients received a hamstring or patellar tendon autograft.

All subjects were examined according to a standard protocol including range of motion and knee laxity measurements (manual Lachman test, pivot shift test). Although all patients followed the standardized protocol at the clinic, only data related to the laxity measurements were presented in the current study, in accordance with the study aim. The clinical examination was performed prior to the device assessments in all patients, and the injured side was unknown until examination with the arthrometers.

All participants received oral and written information about the study before signing an informed consent. The study was approved by the Swedish ethical review authority with the registration number DNR 2020‐02062 in accordance with the Declaration of Helsinki.

An SSD of ≥3 mm was considered an ACL injury.^4^, ^14^ For the dichotomous variables, SSD of <3 mm was considered as no ACL injury (no) and ≥3 mm was considered to be an ACL injury (yes) for both the GNRB and the KT1000. For the manual Lachman test, 0 to 1 was considered no injury and ≥2 as an ACL injury, both pre‐ and postoperatively.

### GNRB

The test procedure with the GNRB was performed following the instructions recommended for the GNRB by the manufacturer.^4^ The patient was placed in a supine position, and the leg was placed in the device with the knee in 20° flexion. The apex of the patella was palpated on both sides, and a line was drawn horizontally at this level. Hereafter, the leg was placed into the device, loosely buttoning the knee pad correctly and then adjusting the foot, noting the length of the leg on both sides of the foot pad and making sure that the measurements were aligned (Figure 1). Then, the patellar straps were tightened evenly over the patella. The correct placement of the sensor was indicated by the computer when placed on the tibial tuberosity in a 90° angle. The patient was asked to let their arms rest alongside their body. The noninjured side was always tested first. A test round was executed at 150 N after making sure the patient felt safe and comfortable. This was continued with 3 measurements in a row at 200 N. The mean value of the 3 measurements was registered. The patellar pressure was also calculated, reported, and compared between examiners to minimize bias.

**FIGURE 1:**
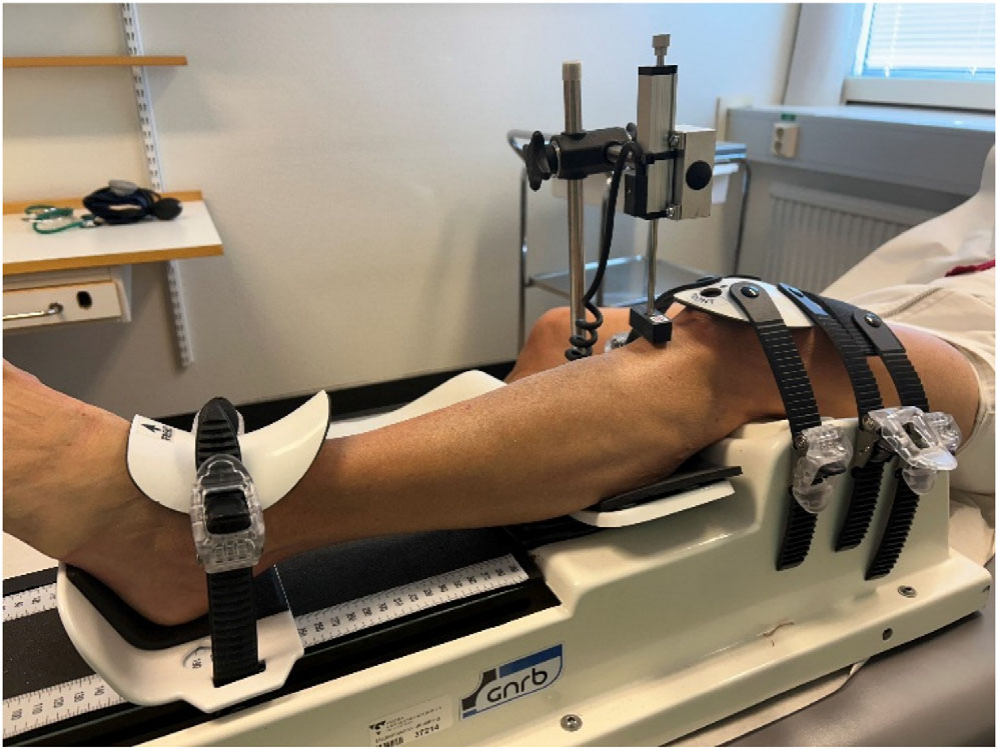
Photograph of the GNRB setup during examination.

The device was used by 2 examiners (S.L.B., N.S.), one with many years of experiences with both the clinical examination and arthrometers (mainly KT1000 arthrometer) and one with no experience with these devices, but with good clinical experience. Before starting the study, a training session via Skype was conducted by the manufacturer, and both examiners practiced the procedure on 10 healthy subjects and read the instructions several times. When training was completed, all remaining questions were addressed with the manufacturer and clarified.

The participants were tested twice by each examiner, prior to surgery and postoperatively. The examiner who started the measurement with the GNRB device before surgery was randomly chosen, and they then alternated during the follow‐up examination. The results were blinded for the next examiner until both exams had been performed. Further, the patient's leg was taken out of the device before the next examiner came into the room.

### KT1000

Examination with the KT1000 arthrometer was performed only by the experienced examiner (examiner 2) at 134 N and manual maximum test, as previously described.^15^ The patient was placed in a supine position, and a thigh support kept the knees in 30° of flexion. A thigh strap and a footrest ensured that the legs stayed in a neutral position. The device was then placed on the lower leg, and 3 measurements were performed on each knee at 134 N and manual maximum test. The average value was registered. The device was calibrated to zero before every test session, and the noninvolved leg was always tested first.

### Manual Lachman

The clinical examination was performed by examiner 2. The manual Lachman test was performed with the patient in a supine position with the arms in a relaxed position alongside the body. The test was performed first on the noninvolved leg and then on the involved side. The results were graded from 0 to +3. Lachman's grade 0 to +1 was considered normal and ≥+2 was diagnosed as an ACL tear. The results were compared with the results of the physician's examination to reduce bias.

The measurements obtained from the GNRB device were automatically calculated and saved onto the laptop connected to the GNRB device with the patient's identification number. At the follow‐up, all patients’ results were compared with their own previous values measured and were shown to the respective patients (Figure 2).

**FIGURE 2:**
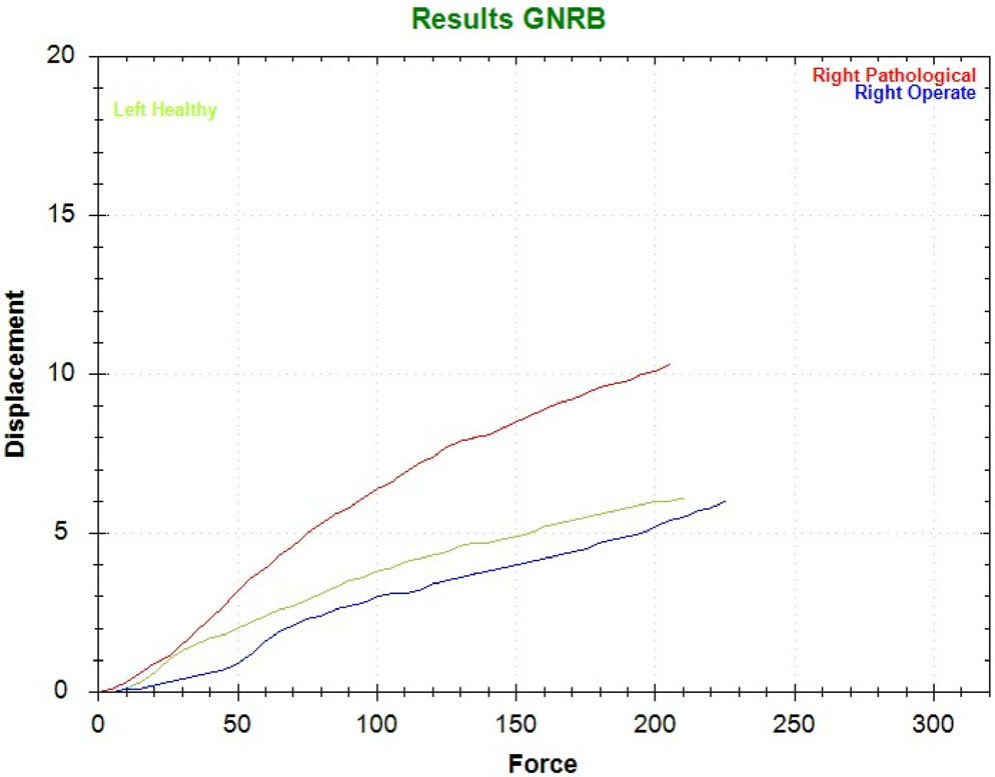
Diagram of the GNRB device for the measurements provided on the healthy, injured, and operated sides. The difference between healthy and operated is less than 3 mm. Displacement presented in millimeters.

Comparison was made between measurements obtained by the examiners on the 2 test occasions and with the results from the KT1000. Also, these data were compared with the results from the manual Lachman test. The KT1000 and clinical examination were conducted by only 1 examiner for 2 reasons: the accuracy of the KT1000 has been shown to increase with experience, and the aim of the study was not to compare the results of KT1000 between 2 examiners.

### Statistical Analysis

Descriptive data are presented as mean (±standard deviation) and median (range) when applicable. The population was normally distributed for age and body mass index. Paired t‐test was used to identify differences between examiners, and interclass correlation (ICC) was used to evaluate inter‐reliability. The ICC value was graded as poor (<0.5), moderate (0.5‐0.75), good (0.75‐0.9), and excellent (>0.9).^16^ The ICCs were calculated with 95% confidence intervals. Sensitivity was calculated before reconstruction for both examiners for the GNRB and for KT1000 for examiner 2. Specificity could not be calculated as all subjects had an ACL injury.

In the power analysis, it was estimated that there would be a difference between examiners with the GNRB of 1 mm and a standard deviation of 1 mm. Seventeen patients were needed in each group to reach a power of 80%, with a *P* value of .05. To take height for dropouts, the study aimed to include 20 patients.

## RESULTS

In total, 27 participants were screened by physicians at the clinic, and 22 underwent the preoperative assessment. Of the 27 that were screened, 4 were excluded for reasons presented in Figure 3, and 1 ended up not having the ACL reconstruction. One exited the study postoperatively based on discomfort when using the GNRB device the first time. Seventeen participated in the follow‐up (Figure 3).

**FIGURE 3:**
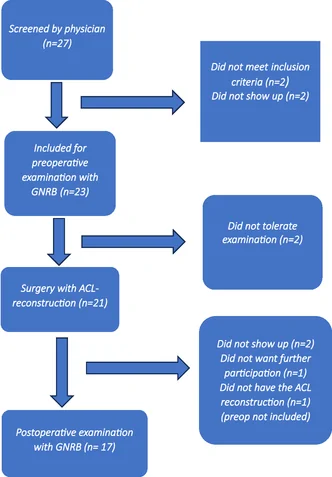
Flowchart of participants and reasons for exiting the study. (ACL, anterior cruciate ligament.)

All details of patient demographics are presented in Table 1. The mean follow‐up time was 10.5 months, and it is noteworthy that most procedures were suspended during the COVID pandemic, which influenced the time to follow‐up. During the waiting period, all participants were advised to continue their rehabilitation (Table 1).

**TABLE 1:** Demographic Data of Study Participants

Number of patients	20
Age (years) Mean (SD) Range	34.1 (11.5) 18‐56
Sex (male/female)	12/8
Injured side (right/left)	10/10
Height (cm) Mean (SD) Range	174 (7.7) 162‐190
Weight (kg) Mean (SD) Range	84.1 (13.2) 65‐115
BMI Mean (SD) Range	27.7 (3.8) 23.1‐36.3
Diagnosed with MRI	16
Concomitant meniscal injury	14
Follow‐up time (months) (time from surgery to postoperative assessment) Mean (SD) Range	10.5 (3.4) 6‐18

BMI, body mass index; cm, centimeter; kg, kilograms; MRI, magnetic resonance imaging; SD, standard deviation.

A comparison of the GNRB and the KT1000 is presented in Table 2, both pre‐ and postoperatively. Both examiners 1 and 2 diagnosed the ACL injury on the group level with the GNRB device (SSD 4.8 and 4.4, respectively). Correspondingly, the experienced examiner diagnosed the ACL injury using the KT1000 arthrometer (SSD 4.5 at 134 N and 4.1 at manual maximum test, respectively) (Table 2). Preoperatively, no difference in displacement and SSD could be found between GNRB and KT1000 values except for the healthy knee at 134 N (*P* = .02). Postoperatively, there was a statistically significant difference between the examiners; however, when comparing the SSD, both examiners had mean values well below the threshold (SSD 1.5 and 1.0, respectively, *P* = .20) (Table 2). Examiners 1 and 2 had similar results when comparing the mean SSD values, although examiner 1 had a wider range both pre‐ and postoperatively (Figure 4).

**TABLE 2:** Mean Values of Examination With the GNRB and KT1000 and Patellar Pressure Pre‐ and Postoperatively

	**GNRB Ex 1**	**GNRB Ex 2**	***P*** **Value Ex 1 vs 2**	**Patellar Pressure (N)** **Ex 1**	**Patellar Pressure (N)** **Ex 2**	***P*** **Value** **Ex 1 vs 2**	**KT 134 N**	**KT MMT**
Involved preop Mean mm (SD) Range	11.9 (2.7) 8.5‐16.8	11.6 (2.8) 7.4‐17	n.s. (.48)	69.3 (8.2) 57.7‐87.0	67.1 (9.9) 53.7‐89.0	n.s. (.2)	10.2 (3.8) 5‐19	11.1 (3.9) 6‐20
Healthy preop Mean mm (SD) Range	7.1 (1.3) 5.4‐10.3	7.2 (1.3) 5.2‐7.2	n.s. (.75)	69.6 (7.1) 59.7‐83.3	68.0 (8.3) 53.7‐82.0	n.s. (.4)	5.7 (3.0)∗ 2‐14	7.1 (4.2) 2.5‐18
Side‐to‐side diff preop (N) Mean mm (SD) Range	4.8 (3.0) 0.4‐10.1	4.4 (2.4) 0.6‐8.8	n.s. (.36)				4.5 (2.3) 0.5‐13	4.1 (3.7) −7.5 to 13
Mean diff Ex 1 vs 2 (SD)	0.38 (1.8)							
Involved postop Mean mm (SD) Range	8.8 (2.3) 5.4‐13	7.7 (1.9) 5‐11.8	***P*** ** = .002**	68.1 (7.0) 58.3‐80.0	70.1 (8.6) 55.3‐86.0	n.s. (.3)	8.5 (2.9) 4‐13.5	9.7 (4.3) 4‐23
Healthy postop Mean mm (SD) Range	7.3 (1.4) 4.9‐11.6	6.8 (1.5) 4.5‐9.8	n.s. (.07)	69.3 (9.2) 56.0‐89.7	70.5 (8.8) 57.3‐87.3	n.s. (.7)	6.2 (2.7) 2.5‐14	7.4 (3.4) 3‐17
Side‐to‐side diff postop Mean mm (SD) Range	1.5 (2.6) −2 to 6.8	1.0 (2.2) −3.2 to 4.5	n.s. (.20)				−2.3 (2.7) −1 to 9	−2.3 (3.0) −2 to 8.5
Mean diff Ex 1 vs 2 mm (SD)	0.52 (1.6)							

*Note*: No significant differences were found in terms of the patellar pressure between the examiners both pre‐ and postoperatively. *P* values < .05 were considered significant. The significant values are shown in bold.

diff, difference; Ex, examiner; MMT, manual maximum test; n.s., nonsignificant; N, Newton; postop, postoperatively; preop, preoperatively; SD, standard deviation.

∗ KT 134 vs examiner 2, *P* = .02.

**FIGURE 4:**
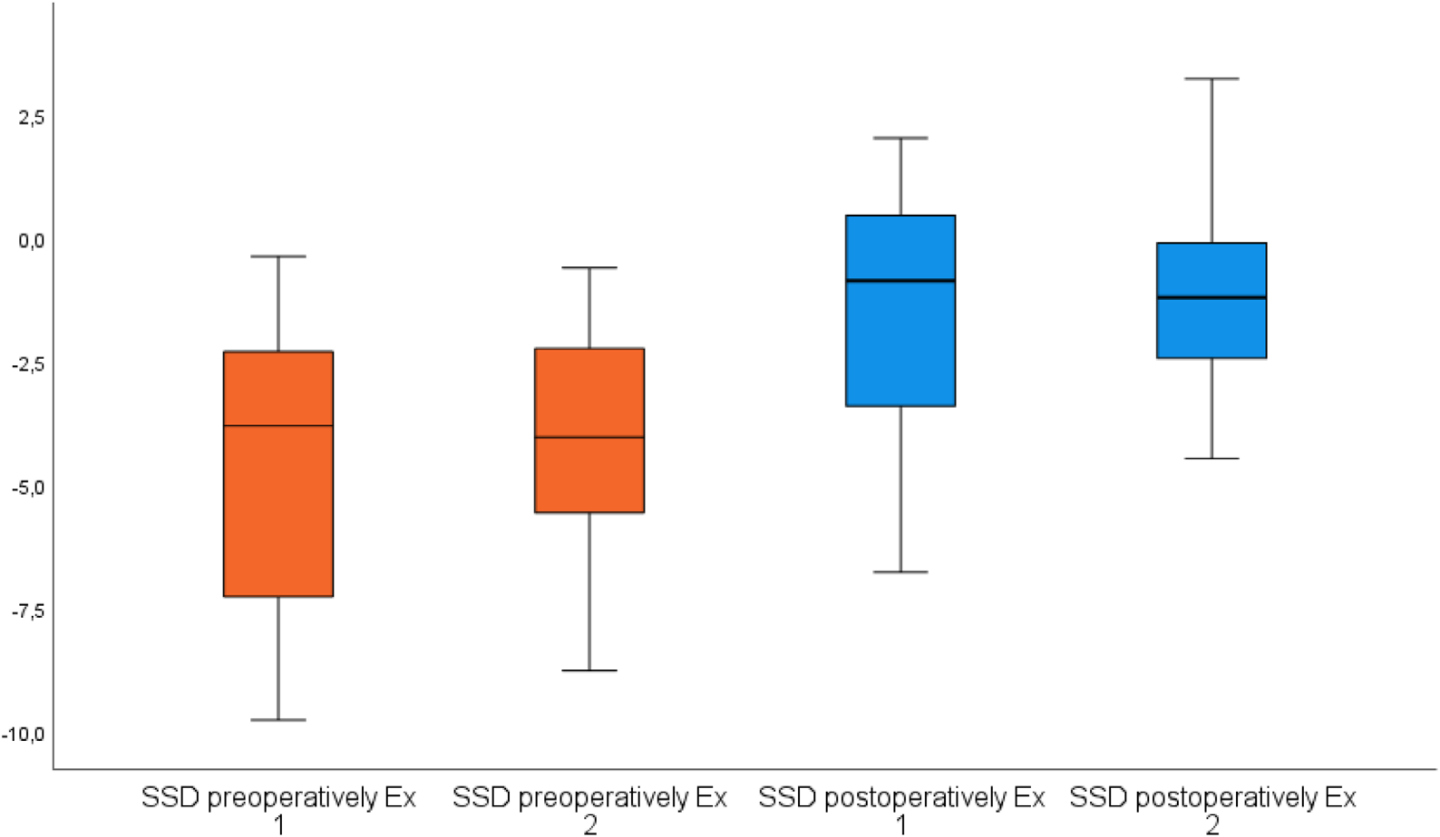
Comparison of the SSD mean values for examiners 1 and 2 pre‐ and postoperatively using the GNRB. (Ex, examiner; SSD, side‐to‐side difference.)

However, for the dichotomous values, the ACL injury was only found in 12/20 and 13/20, respectively, for the GNRB, whereas the KT1000 arthrometer found 19/20 (Table 3). When looking at the specific patients, the examiners agreed in 11 out of the 12 and 13 positive findings. In total, disagreement was seen in 4 specific patients. Similarly, postoperatively, the GNRB identified 12/17 and 13/17 as “noninjured” or “sufficient” reconstructions (SSD ≤ 3 mm) (Table 3), and agreement was seen in 13 out of the 17 participants.

**TABLE 3:** Dichotomous Classification of ACL Injury

GNRB preop examiner 1 Yes/no	12/8
GNRB preop examiner 2 Yes/no	13/7
KT1000 134 N preop Yes/No	19/1
KT1000 MMT preop Yes/no	17/3
Positive manual Lachman (≥+2) preop involved side Yes/no	16/4
Positive manual Lachman (≥+2) preop noninvolved side Yes/no	0/20
GNRB 6 months examiner 1 Yes/no Missing values	5/12 3
GNRB 6 months examiner 2 Yes/no Missing values	4/13 3
KT1000 134 N 6 months Yes/no Missing values	7/10 3
KT1000 MMT 6 months Yes/no Missing values	8/9 3
Positive manual Lachman (≥+2) postop, involved side Yes/no Missing values	2/15 3
Positive manual Lachman (≥+2) postop, noninvolved side Yes/no Missing values	0/17 3

*Note*: GNRB and KT1000: SSD < 3.0 mm classified as no ACL injury and ≥3.0 mm as ACL injury. Manual Lachman: grade 0 to (+)1 classified as no ACL injury and greater than 2 as ACL injury.

ACL, anterior cruciate ligament; MMT, manual maximum test; N, Newton; postop, postoperatively; preop, preoperatively.

The ICC was higher for the involved leg. The highest agreement was seen for the involved side postoperatively, with 0.85. The ICC was the lowest for the healthy side (0.48 and 0.69, respectively). For specific details, see Table 4. The sensitivity for the GNRB was 60% for examiner 1 and 65% for examiner 2. For the KT1000, the sensitivity was 95%.

There was no significant difference between the mean patellar pressure between the examiners (*P* > .05) in any of the groups; however, both examiners had a wide range both pre‐ and postoperatively (Table 2, Figure 5a,b).

**TABLE 4:** Interclass Correlation and Confidence Intervals for Examiners 1 and 2 Pre‐ and Postoperatively

	**Involved Side Preop**	**Healthy Side Preop**	**Difference Healthy/Involved Preop**	**Involved Side Postop**	**Healthy Side Postop**	**Difference Healthy/Involved Postop**
ICC 95% CI interval	0.80 0.55‐0.91	0.48 0.06‐0.76	0.78 0.52‐0.91	0.85 0.63‐0.94	0.69 0.32‐0.87	0.77 0.48‐0.91

CI, confidence interval; ICC, interclass correlation coefficient; Preop, preoperatively; Postop, postoperatively.

**FIGURE 5:**
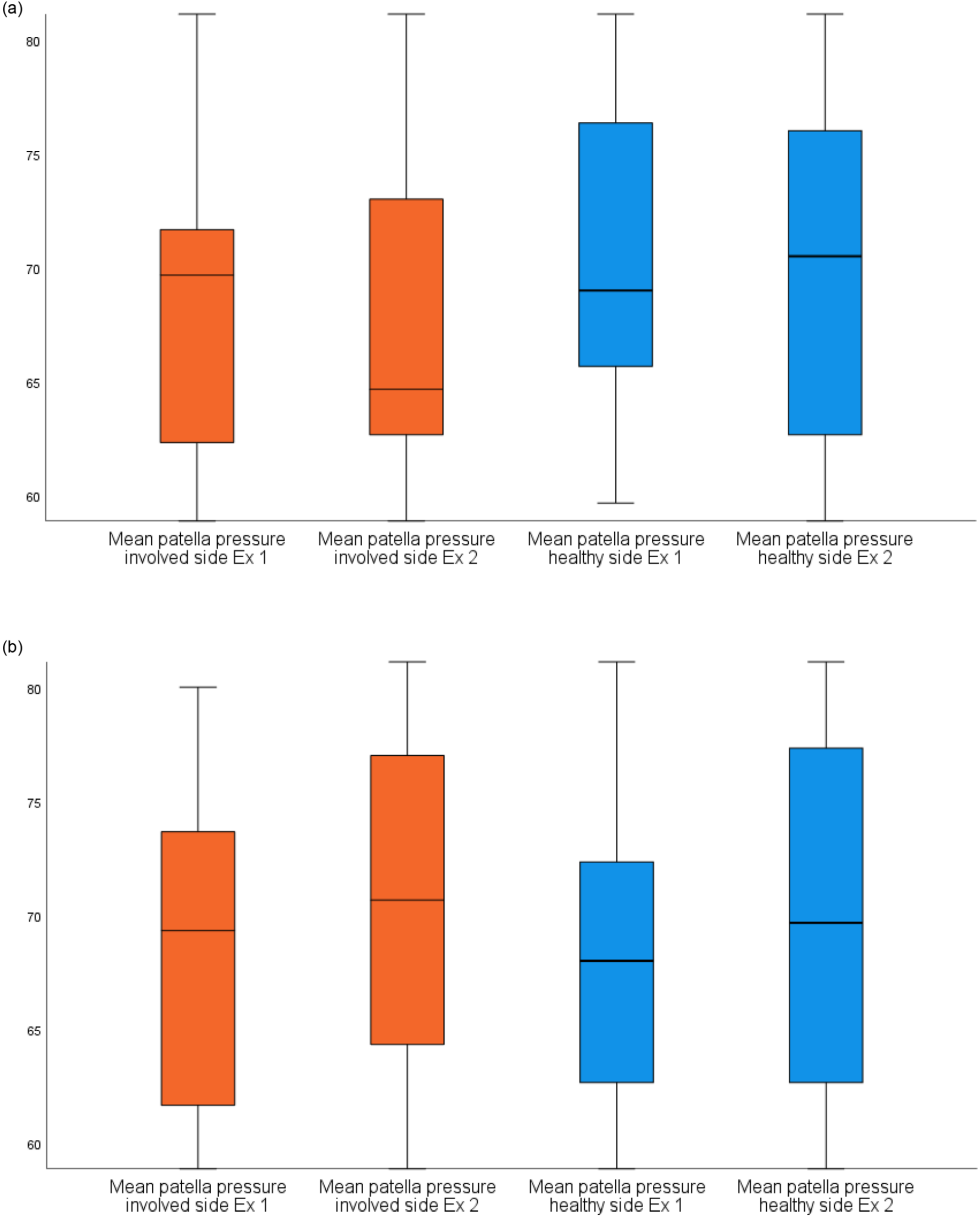
(a) Patellar pressure preoperatively for examiners 1 and 2. (b) Patellar pressure postoperatively for examiners 1 and 2. (Ex, examiner.)

## DISCUSSION

The most important finding in this study was that although the ICC was good for the involved side, the GNRB only identified 12/20 and 13/20 with an ACL injury, respectively, for the 2 examiners, whereas the KT1000 arthrometer identified 19/20. Further, the sensitivity for the GNRB in the current study was 60% and 65% preoperatively for the 2 examiners, respectively.

As for comparison with the KT1000 arthrometer, examiner 2 had many years of experience with the device, something that is known to influence the results.^4^, ^15^, ^17^ With the GNRB, both examiners had limited experience. For this reason, comparisons were only made with KT1000 for examiner 2. Both the GNRB and the KT1000 were able to identify the ACL injury on a group level when compared on a mean value basis, but the GNRB only correctly identified two‐thirds of the injured patients preoperatively. A preoperative sensitivity of 60% to 65% as found in the present study is lower than previous studies.^4^, ^18^ Further, postoperatively, GNRB still identified 4 and 5, respectively, with a laxity above the cutoff, indicative of an ACL injury, whereas the clinical examination ruled 15/17 as sufficient. Even though an SSD of 3 mm has been suggested by the manufacturer,^4^ others have suggested that this limit might be too narrow.^10^, ^19^ As for the current study, both examiners had similar results both for mean and SSD values, although the ranges differed. Thus, the present study suggests that although the GNRB offers good inter‐reliability and ICC, it might not be reliable enough to replace the clinical examination or the magnetic resonance imaging in diagnosing an ACL tear in each individual patient. The same limitation has been found for the KT1000;^15^ hence, arthrometers offer an additional contribution to clinical examination and magnetic resonance imaging, especially in research studies.

The ICC values in the current study are higher than in some of the previous studies; however, comparison between the GNRB and the KT1000 has found that GNRB is superior.^4^, ^11^, ^19^ Magdic et al. found a higher ICC on both sides in healthy active participants; however, the patellar pressure was supervised very closely.^20^ This could possibly point in the direction that the applied patellar pressure is of higher importance than first described. Although the device was easy to learn, both examiners found it difficult to apply the correct patellar pressure. Additionally, the patellar pad and straps sometimes did increase the patellar pressure without obvious reason. This has been discussed before, and the reasons for this are not known, although it has been suggested that the patient's ability to relax, body mass index, and patellar straps’ positioning would impact this and thus also impact the results.^9^, ^10^ In the current study, no significant difference was found between the applied patellar pressure between the examiners. However, there was a wide range for both examiners, indicating the difficulties mentioned above. Further, the current study aimed to examine the use of the GNRB in the everyday setting, as different examiners will be performing the measurements; thus, a difference in the patellar pressure is to be expected.

The manual Lachman test found that 15/17 had sufficient stability postoperatively, although it should be mentioned that the expectation of a successful result might influence the clinical tests. Further, the manual Lachman considers the presence of a firm endpoint even with increased laxity, something that might not be considered by the arthrometers. Previously, the Lachman test has been found inferior to the GNRB,^6^ whereas in the current study, the clinical testing was performed before the examination with the devices and found more injuries than the GNRB. Until the measurement with the arthrometers, the injured side remained unknown to the examiners.

The use of the GNRB arthrometer takes extra time for setup and examination, is heavy, difficult to move and transport, and has the need for an attached computer. This, in comparison with the KT1000, limits the everyday use of the device.

### Limitations

This study is not without limitations. First, the KT1000 was used only by examiner 1, which weakens the comparisons in this study; however, the lack of experience has been shown to influence the results.^4^, ^15^, ^17^ The manual Lachman was not blinded postoperatively, which could add bias to this finding. Further, the relatively small study sample increases the risk of type 2 error. Larger cohorts are needed to validate the current findings. There was no control of the patellar pressure added to the same patient between examiners, which could have influenced the results. However, the GNRB device is presented as a diagnostic tool, and the current study aimed to analyze it in a setting similar to that of everyday work, including experienced and novice examiners, different patellar pressures, and possibly different rotations of the tibia for each examination.

## CONCLUSIONS

In this study, the GNRB had a high inter‐reliability, even with limited experience with the device. However, it was inferior to the KT1000 in correctly identifying ACL deficiency preoperatively in patients with ACL injury.

## DISCLOSURES

The authors (S.L.B., J‐T.K.) declare the following financial interests/personal relationships which may be considered as potential competing interests: S.L.B. reports financial support provided by NU Hospital Group. J‐T.K. reports a relationship with CONMED Sweden that includes speaking and lecture fees; reports a relationship with Swedish National Knee Register that includes board membership. The other author (N.S.) declares that she has no known competing financial interests or personal relationships that could have appeared to influence the work reported in this article.
